# Application of multimodal nanotechnology and standardized nursing management in ventricular arrhythmia

**DOI:** 10.1186/s12938-026-01571-0

**Published:** 2026-04-17

**Authors:** Xueqin Ding, Lianghong Zhang, Fangming Guo, Xuhong Pan, Xiaona Wei, Lingfei Su, Ling Wu, Li Min, Ming Zhang, Li Han

**Affiliations:** 1 Cardiac Intensive Care Unit, Yantaishan HospitalYantai City, Binzhou Medical University, Yantai, 264003 Shandong China; 2 Cardiovascular Department, First District, The People’s Hospital of Zhaoyuan City, Yantai, 265400 Shandong China; 3 Department of Medical Imaging, Yantaishan Hospital, Yantai, 264003 Shandong China; 4 Nursing Department, Yantaishan Hospital, Yantai, 264003 Shandong China; 5 Department of Medical Insurance Management, Yantaishan Hospital, Yantai, 264003 Shandong China; 6 Department of Medical Instruments, Yantaishan Hospital, Yantai, 264003 Shandong China; 7 Department of Management, Yantai Huitong Jiaren Medical Technology Co., Ltd., Yantai, 264000 Shandong China; 8 Department of Hospital Management, Yantaishan Hospital, Yantai, 264003 Shandong China No. 10087 Science and Technology Avenue, Laishan District,; 9 Cardiac Intensive Care Unit, Yantaishan Hospital, Yantai City, No. 10087 Science and Technology Avenue, Laishan District, Yantai, 264003 Shandong China

**Keywords:** Ventricular arrhythmia, Multimodal nanotechnology, Precision medicine, Nursing management, Integrated care, Translational barriers

## Abstract

Ventricular arrhythmias, particularly ventricular fibrillation, remain a leading cause of sudden cardiac death and pose persistent clinical challenges due to limitations in targeted therapy and the complexity of arrhythmogenic substrates. multimodal nanotechnology has emerged as a promising platform enabling integrated diagnostic and therapeutic functions; however, direct evidence for its application in cardiology remains limited compared to oncology, and significant translational barriers persist. Concurrently, comprehensive nursing and standardized management are critical for optimizing patient outcomes through continuous monitoring, timely intervention, and holistic care. This review critically synthesizes current evidence on multimodal nanotechnology applications in ventricular arrhythmia management, distinguishing between established cardiovascular findings and conceptual extrapolations from other fields. It analyzes technological advantages alongside cardiac-specific limitations—including myocardial targeting challenges, pro-arrhythmic risks, and regulatory complexity. Furthermore, it examines how nanotechnology-based therapies will impact nursing workflows, monitoring protocols, and competency requirements, proposing a framework for integrating these innovations with standardized nursing management. By bridging cutting-edge technological advances with clinical nursing strategies, this article provides a theoretical foundation and practical guidance for fostering multidisciplinary collaboration and advancing comprehensive, patient-centered treatment paradigms for ventricular arrhythmias.

## Introduction

Ventricular arrhythmias, particularly ventricular fibrillation (VF), constitute a critical and life-threatening subset of cardiovascular disorders. Characterized by sudden onset and high mortality, VF remains a predominant mechanism of sudden cardiac death worldwide, underscoring an urgent need for innovative therapeutic strategies to improve survival and clinical outcomes [1]. The management of these acute arrhythmias poses significant challenges to conventional approaches, which primarily rely on pharmacological agents and implantable cardioverter-defibrillators (ICDs). These strategies are often constrained by limitations including delayed diagnosis, lack of tissue specificity leading to off-target effects, suboptimal long-term control of arrhythmogenic substrates, and the intrinsic pro-arrhythmic potential of many antiarrhythmic drugs. The complex pathophysiology involving electrophysiological and structural remodeling in the ventricular myocardium—including fibrosis, gap junction dysfunction, and ion channel abnormalities—necessitates the development of advanced theranostic modalities capable of enabling precise, timely, and effective intervention.

In response to these challenges, multimodal nanotechnology has emerged as a transformative frontier in cardiovascular biomedicine. This approach leverages integrated nanoplatforms that converge diagnostic and therapeutic functions at the nanoscale. By exploiting the unique physicochemical properties of nanomaterials—such as high surface-to-volume ratio, tunable size and morphology, and surface functionalizability—these systems can be engineered for targeted accumulation within arrhythmogenic cardiac tissues. Multimodal nanotechnologies facilitate a synergistic combination of capabilities including high-sensitivity imaging, controlled drug or gene delivery, and real-time biosensing. This integrated theranostic paradigm holds promise for revolutionizing ventricular arrhythmia management by enabling early detection of pro-arrhythmic substrates, spatially targeted therapy with minimized systemic exposure, and dynamic treatment response monitoring, thereby paving the way for personalized medicine. However, it is critical to acknowledge that much of the foundational research in multimodal nanotechnology has been conducted in oncology, where tumor biology—characterized by enhanced permeability and retention effects and distinct microenvironmental features—differs fundamentally from cardiac tissue. Direct translation of these findings to ventricular arrhythmia management requires rigorous validation and adaptation to address cardiac-specific physiological and pathological characteristics.

The successful clinical translation of such advanced technologies extends beyond device and drug development to encompass holistic patient care. A comprehensive management framework is essential, integrating cutting-edge interventions with standardized nursing protocols and systematic care pathways. Structured nursing care is pivotal across the patient journey, encompassing critical aspects such as peri-procedural monitoring, prevention of complications (e.g., infection, thrombosis), patient education on device management and lifestyle modification, and provision of psychosocial support. The introduction of nanotechnology-based therapies will necessitate a corresponding evolution in nursing practice, including new competencies in nanomedicine administration, monitoring for novel adverse effect profiles, and patient education regarding these innovative treatments. Concurrently, the implementation of evidence-based, standardized management protocols ensures consistency, enhances patient safety, improves workflow efficiency, and facilitates the seamless integration of novel technologies into routine clinical practice. The synergy between targeted nanotechnology-based solutions and a robust, patient-centered care system is fundamental to achieving superior clinical outcomes, enhancing quality of life, and optimizing healthcare resource utilization.

Accordingly, this review aims to systematically synthesize contemporary advances in multimodal nanotechnology applications for the diagnosis and treatment of ventricular arrhythmias, with a focused discussion on VF while critically distinguishing between established cardiovascular evidence and conceptual extrapolation from other fields. It further examines the integral role of comprehensive nursing care and standardized management strategies tailored to this high-risk population, including practical implications for nursing workflows and competency development. By critically evaluating the convergence of technological innovation and optimized care delivery, this article seeks to bridge the gap between foundational research and clinical implementation. The ultimate goal is to foster interdisciplinary collaboration among cardiologists, biomedical engineers, nursing professionals, and healthcare systems engineers, thereby contributing to the development of more effective, safe, and patient-centric paradigms for combating ventricular arrhythmias and improving cardiovascular health.

## Basic principles and current development status of multimodal nanotechnology

### Definition and classification of multimodal nanotechnology

Multimodal nanotechnology refers to the integration of multiple diagnostic and therapeutic functions within a single nanoscale platform, enabling their simultaneous or sequential application. Its core concept lies in the convergence of materials science and biomedicine, aiming to construct multifunctional nanodevices capable of performing tasks such as targeted imaging, controlled drug delivery, and photothermal/photodynamic therapy. A hallmark of this technology is the combination of diagnostic and therapeutic functions—known as “theranostics”—thereby enabling precise disease detection, real-time monitoring, and personalized treatment. This integration improves clinical outcomes by enhancing specificity, reducing systemic toxicity, and leveraging synergistic therapeutic effects. For example, nanoparticles can be engineered to simultaneously carry contrast agents for magnetic resonance or fluorescence imaging along with therapeutic agents, or to possess photothermal ablation capabilities, offering a comprehensive approach to disease management [2, 3].

Multimodal nanosystems can be primarily classified from two dimensions: material composition and functional modality. From a materials perspective, commonly used nanomaterials include: (1) metal-based nanoparticles (e.g., gold, silver, iron oxide nanoparticles), prized for their unique optical properties, good biocompatibility, and ease of surface modification, making them ideal candidates for imaging contrast enhancement and photothermal therapy. For instance, gold nanoparticles exhibit strong surface plasmon resonance effects, enabling efficient photothermal conversion for combined imaging and thermal ablation therapy [4]. (2) Lipid-based carriers (e.g., liposomes and lipid nanoparticles), offering good biocompatibility and the ability to co-encapsulate both hydrophilic and hydrophobic drugs, serving as versatile platforms for drug delivery and imaging agent co-loading. (3) Polymeric nanoparticles (composed of natural or synthetic polymers), characterized by tunable physicochemical properties, controllable release kinetics, and ease of functionalization, making them suitable for targeted delivery and multimodal imaging applications [5].

From a functional perspective, multimodal nanosystems are classified based on their integrated capabilities, primarily including imaging, drug delivery, and therapy. Imaging functionalities encompass techniques such as magnetic resonance imaging, fluorescence imaging, photoacoustic imaging, and surface-enhanced Raman spectroscopy. Nanoparticles can be designed to co-load MRI contrast agents (e.g., gadolinium or iron oxide) and fluorescent dyes, achieving dual-modality imaging that combines high spatial resolution with molecular specificity [6, 7]. Drug delivery functions involve the encapsulation or conjugation of therapeutic agents, enabling targeted, controlled, and sustained release at the disease site, thereby minimizing off-target effects and improving efficacy [2, 5]. Therapeutic modalities integrated into multimodal nanoplatforms include photothermal therapy, photodynamic therapy, and emerging approaches like piezoelectric therapy, which utilizes mechanical stimuli to generate local electric fields and reactive oxygen species for tissue ablation [8]. The combination of these functions within a single nanoplatform exemplifies the multimodal approach, facilitating the development of synergistic treatment regimens capable of overcoming the limitations of monotherapies.

### Technological development history and recent advances

The developmental trajectory of nanotechnology in treating cardiovascular diseases, particularly ventricular arrhythmias, mirrors the broader evolution in biomedical fields from simple nanoparticle applications toward sophisticated, multifunctional integrated platforms. Initially, nanotechnology in cardiovascular medicine focused primarily on enhancing drug delivery and imaging capabilities by exploiting the unique physicochemical properties of nanoparticles, such as their high surface area-to-volume ratio and modifiable surfaces. Over time, the field has pivoted toward multimodal nanotechnologies that combine diagnostic and therapeutic functions (i.e., theranostics), enabling simultaneous imaging and treatment within a single nanosystem. This integration addresses the complexity of cardiovascular pathologies by facilitating precise localization, controlled drug release, and real-time monitoring of therapeutic efficacy. A breakthrough in multimodal integration is exemplified by the development of nanoparticles capable of carrying multiple imaging agents or therapeutic compounds, which enhance disease-targeting specificity and sensitivity while reducing systemic toxicity. For instance, dendrimer-based nanoprobes can self-assemble into nanomicelles that provide enhanced MRI contrast and near-infrared fluorescence imaging capabilities. This has demonstrated superior tumor detection in oncology, suggesting analogous potential for cardiac tissue imaging and therapy, though direct validation in cardiac models remains limited [6]. Similarly, the application of pegylated crosslinked hyaluronic acid nanoparticles has proven the feasibility of co-encapsulating MRI and optical imaging agents, achieving multimodal diagnostic capabilities with improved biocompatibility and imaging performance [7].

Recent innovations have also emphasized the design of nanomaterials with intelligent surface modifications and stimuli-responsive properties, advancing the field of “smart” nanotechnology. Surface functionalization strategies—including ligand conjugation and polymer coatings—enhance targeting precision toward pathological cardiac tissues while reducing off-target effects and immune clearance. Furthermore, incorporating responsive mechanisms (e.g., pH-, temperature-, or enzyme-triggered drug release) enables controlled and site-specific therapeutic action. These advances are critical for addressing the dynamic and heterogeneous microenvironment of cardiac arrhythmias, where localized delivery and real-time responsiveness can significantly improve treatment outcomes. For example, the development of programmable multimodal optothermal platforms has enabled the precise manipulation of synthetic nanoparticles and biological cells using low-power lasers, demonstrating potential for autonomous control and targeted intervention at the cellular level [9]. Such technologies could potentially be adapted to modulate cardiac electrophysiological activity or to deliver antiarrhythmic agents with spatiotemporal precision.

Beyond material design, the integration of Artificial Intelligence (AI) with nanotechnology has emerged as a transformative approach to enhance image-guided therapy and precision medicine. AI algorithms improve image acquisition, processing, and interpretation, thereby augmenting the diagnostic accuracy and therapeutic guidance provided by multimodal nanoprobes. This synergy is particularly relevant in cardiovascular applications, where complex anatomical and functional data must be analyzed rapidly and accurately to guide interventions. Furthermore, the introduction of green nanotechnology aims to address concerns regarding nanoparticle toxicity and environmental impact by promoting the synthesis of sustainable and biocompatible nanomaterials through eco-friendly methods. This approach ensures safer clinical translation and long-term patient safety, which is paramount in the management of chronic cardiovascular diseases [3].

Collectively, these technological advancements reflect the maturation of nanotechnology from single-function nanoparticles toward integrated, multifunctional platforms that combine advanced materials engineering, intelligent responsiveness, and AI-driven precision. Although much pioneering work has been validated in oncology and other systemic diseases, the underlying principles require careful adaptation to cardiovascular medicine, including the management of ventricular arrhythmias. Key differences—such as the absence of an EPR effect in the myocardium, the contractile and electrophysiologically active nature of cardiac tissue, and the need for rapid therapeutic action in acute arrhythmias—mandate disease-specific validation. Ongoing research focusing on the convergence of multimodal imaging, targeted drug delivery, and intelligent control mechanisms promises to overcome current therapeutic limitations, enabling highly personalized and effective interventions for patients with arrhythmias. The remaining challenges lie in optimizing biocompatibility, minimizing immune reactions, addressing pro-arrhythmic risks, and scaling up production for clinical application [3, 7, 9, 10].

### Advantages and challenges of multimodal nanotechnology

Multimodal nanotechnology offers significant advantages in enhancing the sensitivity, targeting specificity, and therapeutic efficacy for diagnosing and treating diseases, including complex conditions like ventricular arrhythmias. A primary benefit is its ability to integrate multiple diagnostic and therapeutic functions into a single nanoscale platform, enabling simultaneous multimodal imaging and multimodal therapy. For instance, nanoparticles can be engineered to carry various imaging agents—such as MRI contrast agents, near-infrared fluorescent dyes, and radioactive isotopes—thereby providing complementary morphological and functional information that improves diagnostic accuracy and early detection sensitivity [6, 11]. This multimodality enhances the capability to precisely localize pathological sites, monitor therapeutic responses in real-time, and guide interventions with higher spatial and temporal resolution. Furthermore, by functionalizing the surface of nanoplatforms (e.g., conjugating ligands that recognize disease-specific biomarkers), targeted delivery of therapeutic agents can be achieved. This increases drug accumulation at the pathological site while minimizing off-target effects and systemic toxicity [2, 12]. The high surface area-to-volume ratio of nanoparticles facilitates the loading of multiple therapeutic agents, allowing for the co-delivery of combined therapies such as chemotherapy, photothermal therapy, and immunotherapy, thereby enhancing treatment efficacy through synergistic mechanisms [13, 14]. Additionally, nanomaterials can be designed to respond to specific stimuli in the microenvironment (e.g., pH, redox conditions), enabling controlled and sustained drug release that further improves therapeutic outcomes [10, 15].

Despite this promising potential, the clinical translation and application of multimodal nanotechnology face several significant challenges that are particularly acute in the cardiac context. Biocompatibility remains a critical concern, as introducing nanoparticles into the body can provoke immune responses, inflammation, or unintended toxicity, particularly when high doses are required for effective imaging or therapy [3, 11]. In the heart, this risk is compounded by the potential for nanoparticle-cardiac tissue interactions to alter electrophysiological properties, potentially creating pro-arrhythmic substrates through mechanisms such as ion channel interference, oxidative stress, or inflammatory modulation of gap junction function.

Understanding and controlling the in vivo pharmacokinetics and biodistribution of nanoparticles is complex due to their interactions with biological components, which influence their circulation time, accumulation in target tissues, and clearance pathways [4, 16]. Cardiac targeting presents unique anatomical barriers: the myocardial capillary endothelium is continuous and characterized by tight junctions with an approximate diameter of 4 nm. This severely restricts passive extravasation of nanoparticles, meaning that the enhanced permeability and retention (EPR) effect—a cornerstone of tumor-targeted nanomedicine—is minimal or absent in healthy or even infarcted myocardium. Consequently, cardiac delivery relies almost entirely on active targeting strategies, such as receptor-mediated transcytosis, which require highly specific ligand–receptor interactions and are subject to saturation and competition effects. The coronary circulation also presents challenges, including rapid washout and heterogeneous perfusion patterns in diseased hearts.

Potential long-term toxicity and the degradation products of nanoparticles require thorough evaluation to ensure safety in chronic applications. The heart, as a post-mitotic organ with limited regenerative capacity, may be particularly vulnerable to cumulative nanoparticle retention and slow-release toxic effects. Furthermore, the heterogeneity of pathological microenvironments complicates the design of universal nanoplatforms. Arrhythmogenic substrates range from acutely ischemic tissue (with acidic pH, oxidative stress, and inflammation) to chronic fibrotic scars (with dense extracellular matrix and sparse cellularity) [17, 18]. Variations in pH, enzyme expression, immune status, and perfusion can dramatically affect nanoparticle performance and therapeutic efficacy [19, 20]. A nanoplatform optimized for one substrate may fail in another, necessitating tailored designs for specific arrhythmia contexts.

Manufacturing challenges, including the reproducibility, scalability, and quality control of complex multimodal nanoparticles, further hinder clinical adoption [12, 21]. The complexity of multifunctional nanoparticles—with multiple components, surface modifications, and stimuli-responsive elements—exponentially increases manufacturing variability and the difficulty of batch-to-batch consistency. Regulatory pathways for approving multifunctional nanomedicines remain unclear and demanding due to their hybrid nature combining diagnostics and therapeutics. Regulatory agencies currently lack established frameworks for evaluating combination products where the diagnostic and therapeutic components may have interdependent safety and efficacy profiles. To address these challenges, advances in green nanotechnology aim to develop eco-friendly and biocompatible nanoparticles with reduced toxicity profiles. Integration with artificial intelligence and microfluidic technologies holds promise for optimizing nanoparticle design, enhancing targeting precision, and enabling personalized medicine approaches [3, 22].

Importantly, addressing these multifaceted challenges requires not only continued innovation in materials science and biomedical engineering, but also robust clinical infrastructure. The safe and effective implementation of nanotechnology-based therapies will demand comprehensive nursing strategies—including specialized monitoring protocols, patient education frameworks, and adverse event recognition—alongside standardized management systems that ensure consistency and quality across healthcare settings. These clinical support systems, detailed in subsequent sections, are integral to bridging the gap between technological promise and real-world patient benefit.

In conclusion, while multimodal nanotechnology presents transformative advantages in improving diagnostic sensitivity, targeting specificity, and therapeutic efficiency, overcoming challenges related to biocompatibility, pharmacokinetics, toxicity, manufacturing, and regulation is essential to fully realize its clinical potential.

## Application of multimodal nanotechnology in anti-arrhythmia therapy

### Precision diagnosis and imaging technologies

Nanoscale probes have fundamentally advanced the localization of arrhythmogenic foci and the detection of electrophysiological anomalies by enabling molecular-level targeting within cardiac tissues. These probes are engineered to selectively bind to specific biomarkers associated with ventricular arrhythmogenesis, such as angiotensin II type 1 receptors upregulated in infarct scars, matrix metalloproteinases in remodeling myocardium, or connexin 43 in gap junction remodeling. This molecular targeting facilitates the early and precise identification of pathological substrates. Their integration with advanced multimodal imaging techniques—such as magnetic resonance imaging (MRI) and photoacoustic imaging (PAI)—significantly enhances both spatial and functional resolution. While MRI provides high-resolution anatomical and tissue characterization (e.g., scar delineation, fat infiltration), PAI offers high-contrast, real-time visualization of electrophysiological dynamics and metabolic activity based on optical absorption of hemoglobin, lipids, or targeted contrast agents. This synergistic combination allows for a comprehensive, multi-parametric assessment of cardiac structure and function. Consequently, diagnostic precision is markedly improved, enabling more accurate guidance for subsequent targeted therapeutic interventions against ventricular arrhythmias.

### Targeted drug delivery systems

Nanocarrier-based targeted delivery systems represent a paradigm shift in antiarrhythmic pharmacotherapy. They leverage the unique physicochemical properties of nanomaterials to achieve site-specific accumulation and spatiotemporally controlled release of therapeutic agents. The targeting paradigm primarily involves surface functionalization of nanocarriers (e.g., liposomes, polymeric nanoparticles, inorganic nanomaterials) with ligands or antibodies that recognize molecular signatures overexpressed on cardiomyocytes or within arrhythmogenic tissue. However, it is critical to recognize that cardiac targeting differs fundamentally from tumor targeting. The myocardial capillary endothelium is continuous, with tight junctions approximately 4 nm in diameter that severely restrict passive extravasation of nanoparticles. Consequently, the enhanced permeability and retention (EPR) effect—widely exploited in oncology—is minimal or absent in cardiac tissue. This necessitates reliance on active targeting strategies such as receptor-mediated transcytosis, where nanoparticles are actively transported across endothelial cells via specific ligand–receptor interactions.

Active targeting strategies currently under investigation include functionalization with antibodies or peptides targeting:

- Angiotensin II type 1 receptors (AT1R), upregulated in infarct scars and remodeling myocardium.
- Vascular cell adhesion molecule-1 (VCAM-1), expressed on activated endothelium post-ischemia.
- Myosin light chain fragments are exposed in damaged cardiomyocytes.
- Tenascin-C, an extracellular matrix protein upregulated in fibrotic tissue.

A significant innovation lies in the development of stimuli-responsive or “smart” nanocarriers. These systems are designed to release their therapeutic payload in response to endogenous triggers prevalent in the diseased cardiac microenvironment, such as specific pH gradients (acidosis in ischemia), altered redox states (elevated glutathione in oxidative stress), or overexpressed enzymatic activity (matrix metalloproteinases in remodeling myocardium). For instance, mesoporous silica nanoparticles (MSNs) provide a high-surface-area, rigid framework for substantial drug loading and can be gated with molecular caps that disassemble under pathological conditions, optimizing therapeutic efficacy and minimizing premature leakage [23]. Furthermore, the integration of artificial intelligence and computational modeling in nanocarrier design is refining the selection of targeting moieties and predicting drug release kinetics, thereby enhancing delivery precision and addressing challenges posed by tissue heterogeneity [24].

Emerging biomimetic strategies offer promising solutions to the dual challenges of targeting and immune evasion. Cardiac cell membrane-coated nanoparticles represent an innovative approach where nanoparticles are cloaked in membranes derived from cardiomyocytes, inheriting surface proteins that facilitate homing to cardiac tissue while reducing recognition and clearance by the reticuloendothelial system (RES). Studies have demonstrated that cardiac cell membrane-coated mesoporous silica nanoparticles exhibit significantly enhanced distribution to cardiac cells compared to other cell types, with reduced macrophage phagocytosis, offering a potential strategy to overcome RES uptake and enhance myocardial retention.

Pharmacokinetic considerations are paramount for cardiac nanomedicine. Following intravenous administration, nanoparticles face rapid clearance by the RES (primarily liver and spleen), with circulation half-lives often measured in minutes to hours unless surface-modified with stealth coatings such as polyethylene glycol (PEG). Coronary perfusion delivers nanoparticles to the heart, but retention requires successful endothelial transcytosis and avoidance of washout. Myocardial retention kinetics are poorly characterized but are likely influenced by nanoparticle size, surface charge, and the presence of retention-enhancing moieties. Strategies to improve retention include designing nanoparticles that bind extracellular matrix components or undergo cellular internalization.

This nano-engineering approach directly addresses a major limitation of conventional antiarrhythmic drugs: their narrow therapeutic index and propensity for serious extra-cardiac side effects. Encapsulation within nanocarriers shields drugs from premature degradation, alters their pharmacokinetic profile, and can facilitate traversal of biological barriers like the blood-heart barrier. Liposomal formulations, for example, have demonstrated the ability to reduce the cardiotoxicity and systemic adverse effects of potent drugs by allowing for lower effective doses and providing a sustained release profile [25]. Biomimetic strategies, such as the use of cell membrane-coated or bacterial membrane-derived nanovesicles, further enhance biocompatibility and innate targeting capabilities, potentially combining drug delivery with immunomodulatory functions [26]. The controlled-release properties enable maintenance of therapeutic drug concentrations within the myocardium over extended periods, reducing dosing frequency and improving patient adherence [17].

In summary, targeted drug delivery systems based on nanotechnology offer a multifaceted solution to the challenges inherent in antiarrhythmic drug therapy. Through precise targeting mechanisms and controlled release strategies, these nanocarriers enhance drug accumulation in arrhythmogenic cardiac tissue, improve therapeutic efficacy, and minimize adverse effects. However, successful cardiac targeting requires explicit recognition of the unique anatomical and physiological barriers, particularly the absence of EPR effects and the need for active transcytosis. The ongoing advancements in nanocarrier design, including stimuli-responsive features, biomimetic coating strategies, and AI-guided optimization, hold promise for personalized and safer antiarrhythmic treatments. However, clinical translation requires addressing challenges related to biocompatibility, large-scale production, and regulatory approval to fully realize the potential of nanotechnology in managing ventricular arrhythmias [17–27].

### Photothermal and photodynamic therapy assistance

Nanomaterials serve as pivotal therapeutic enhancers in photothermal therapy (PTT) and photodynamic therapy (PDT) for the targeted modulation or ablation of arrhythmogenic substrates. Engineered nanoparticles, such as gold nanorods, platinum nanoshells, or conjugated polymers, possess tunable optical properties that allow them to efficiently absorb near-infrared (NIR) light. This energy is then converted into localized cytotoxic heat (in PTT) or is used to catalyze the generation of reactive oxygen species (ROS) (in PDT). When functionalized for cardiac targeting, these nanoconstructs enable selective intervention at pathological sites while sparing surrounding healthy myocardium.

Recent preclinical studies have begun to provide direct evidence for the feasibility of phototherapy in cardiovascular applications. Wang et al. developed platinum nanoparticle shells (PtNP-shells) with exceptional photothermal conversion efficiency (73.7% at 1064 nm in the NIR-II window) and demonstrated their application in a canine model of ventricular arrhythmias. By precisely manipulating the photothermal effect, they achieved rapid and precise multimodal neuromodulation encompassing neural activation (41.0–42.9 °C) via TRPV1 channels and neural inhibition (45.0–46.9 °C) via TREK1 channels. This NIR-II photothermal modulation enabled bidirectional reversible autonomic modulation and conferred protection against acute ventricular arrhythmias associated with myocardial ischemia and reperfusion injury during interventional therapy. Importantly, the study demonstrated that photothermal neuromodulation of the left stellate ganglion significantly reduced arrhythmia incidence without causing detectable neuronal damage or systemic toxicity over a 30-day follow-up, providing critical proof-of-concept for the safety and efficacy of this approach in a large animal model [28].

Parallel advances in PDT have also emerged. A recent study presented at the American Heart Association described an oxygen-independent PDT nanocomposite (PPSM@CS/DSS) designed to deplete M1 macrophages selectively in the left stellate ganglion following myocardial infarction. This strategy addresses a fundamental limitation of conventional PDT—its dependence on tissue oxygenation—which is particularly problematic in the hypoxic environment of ischemic myocardium. The nanocomposite, activated by 650 nm light, induced apoptosis of pro-inflammatory M1 macrophages without significantly affecting neuronal cells, resulting in reduced left stellate ganglion activity and decreased incidence of malignant arrhythmias in a beagle model. This work demonstrates that oxygen-independent PDT mechanisms can circumvent the oxygenation barrier and enable targeted immunomodulation in cardiac applications [29].

Additionally, Ronchi et al. demonstrated that red-light excitation of conjugated polymers (PCPDTBT) enabled non-genetic optical modulation of human pluripotent stem cell-derived cardiomyocytes. Optical stimulation accelerated calcium dynamics, shortened action potentials, and increased spontaneous beating frequency, with observed antiarrhythmogenic effects. The phototransduction pathway involved Sarco-Endoplasmic Reticulum Calcium ATPase (SERCA) and Na/Ca exchanger (NCX), suggesting that polymer photoexcitation can directly modulate cardiomyocyte electrophysiology without requiring genetic modification [30].

Despite these promising developments, several critical challenges must be addressed before phototherapies can be translated to clinical arrhythmia management:

Oxygen dependence and ROS-mediated injury: conventional PDT relies on oxygen-dependent ROS generation, which may be ineffective in hypoxic or ischemic myocardial tissue. While oxygen-independent strategies offer a potential solution, their safety profile in chronically ischemic or reperfused myocardium requires further evaluation. Moreover, ROS, while therapeutically useful at controlled levels, can cause off-target myocardial injury at higher concentrations, including lipid peroxidation, mitochondrial dysfunction, protein oxidation, and DNA damage. Excessive ROS may compromise contractile function, induce apoptosis in viable cardiomyocytes, or create new arrhythmogenic substrates through oxidative modification of ion channels and gap junctions. Precise spatiotemporal control of ROS generation and rigorous dose optimization are essential to achieve therapeutic effects without unintended myocardial damage.

Selectivity for arrhythmogenic substrates: achieving selective ablation or modulation of arrhythmogenic tissue while preserving normal conduction and contractility requires dual-targeting strategies: (1) molecular targeting of nanoplatforms to pathological tissue (e.g., using ligands specific to infarct scars, fibrotic tissue, or inflamed regions), and (2) precise light delivery confined to the target region. However, arrhythmogenic substrates are often spatially complex, with interdigitated viable and non-viable tissue, making complete selectivity challenging. Thermal or ROS diffusion from the target site may also affect adjacent myocardium, potentially creating new conduction barriers or altering the electrophysiological properties of critical structures.

Light delivery to deep myocardial tissue: unlike superficially located tumors or autonomic ganglia, arrhythmogenic substrates often reside within the thick, contracting ventricular wall. NIR light penetration in myocardial tissue is limited—typically 1–2 cm for NIR-I (650–900 nm) and 2–3 cm for NIR-II (900–1700 nm)—posing challenges for treating mid-myocardial or septal substrates. Cardiac motion further complicates precise light delivery, as the target region may move relative to the light source during the cardiac and respiratory cycles. Potential solutions under investigation include:

- Catheter-based intracardiac light delivery systems that can be positioned adjacent to endocardial targets.
- Implantable miniaturized light sources (e.g., LED arrays) for chronic applications.
- Ultrasound-mediated delivery or photoacoustic-guided targeting to compensate for motion.
- Gating strategies that synchronize light delivery with the cardiac cycle.

Thermal dose optimization: the temperature thresholds for therapeutic effects (e.g., 41.0–42.9 °C for TRPV1 activation, 45.0–46.9 °C for TREK1 inhibition) are narrow, and exceeding these thresholds risks unintended thermal injury. Precise temperature control requires real-time monitoring and feedback systems, which are not yet clinically available for intracardiac applications. Furthermore, the thermal conductivity of perfused myocardium may dissipate heat more rapidly than in non-perfused tissues, potentially reducing efficacy.

**Fig. 1:**
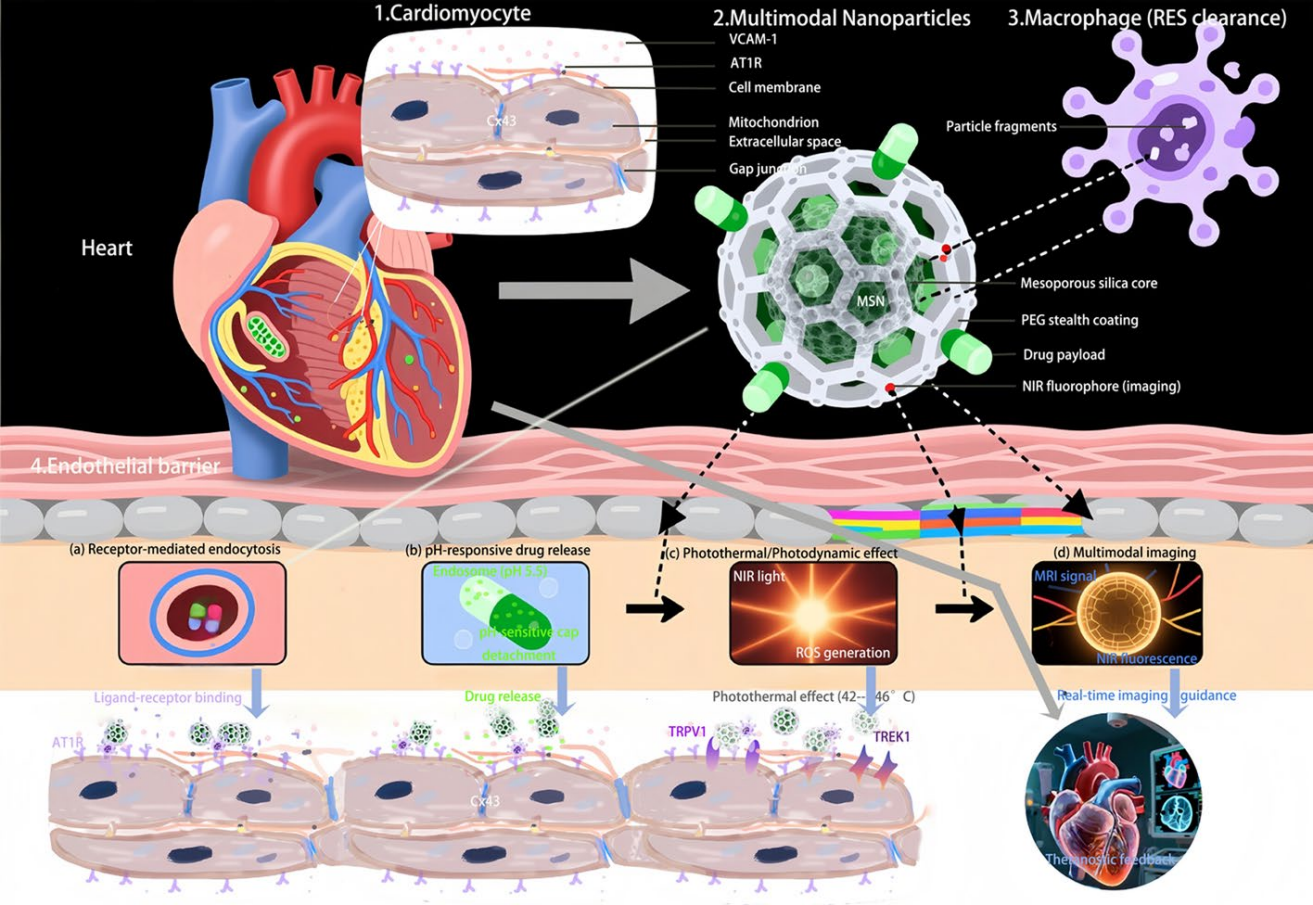
Schematic illustration of a multimodal nanoparticle interacting with a cardiomyocyte. The multifunctional nanoplatform (e.g., mesoporous silica nanoparticle) is engineered with surface functionalization with targeting ligands. Key interactions include: **a** receptor-mediated endocytosis following specific ligand-receptor binding; **b** pH-responsive drug release within the acidic endolysosomal compartment; **c** NIR-triggered photothermal effects for localized ablation or neuromodulation (TRPV1 activation at 41–43 °C, TREK1 inhibition at 45–47 °C); **d** multimodal imaging for real-time treatment guidance

In summary, while nanoparticle-assisted phototherapies offer promising complementary approaches for arrhythmia management—as evidenced by recent preclinical successes in neuromodulation, immunomodulation, and direct cardiomyocyte modulation—substantial technical and biological challenges remain. Rigorous investigation of light delivery systems, thermal/ROS dose optimization, selectivity mechanisms, and long-term safety in clinically relevant models is essential before these approaches can advance to human trials. The field must also establish standardized protocols for light dosimetry, temperature monitoring, and outcome assessment to enable meaningful comparison across studies illustrated in Fig. 1.

### Multimodal nanotechnology-enabled combined therapeutic strategies

The convergence of nanotechnology with established pharmacological and interventional modalities heralds a new era of combinatorial and synergistic treatment paradigms, with proven efficacy in oncology and emergent potential in cardiology, including ventricular arrhythmia management. Nanotechnology provides a versatile platform to unify diagnostic imaging, targeted drug delivery, and physical energy-based therapies (e.g., PTT, PDT) into a single, integrated theranostic system.

This integrated approach is exemplified in oncology by nanoplatforms that combine chemotherapy, gene therapy, and immunotherapy within a single carrier, often co-loaded with imaging contrast agents. For instance, nanoparticles used in transarterial chemoembolization (TACE) for hepatocellular carcinoma not only act as embolic agents but also enable localized, sustained chemotherapeutic release and simultaneous multimodal imaging for precise procedural guidance and treatment monitoring [4]. Similarly, “smart” nanoparticles can be engineered to release multiple therapeutic agents sequentially or simultaneously in response to tumor-specific stimuli, achieving synergistic cytotoxicity while overcoming drug resistance mechanisms [31]. While these oncology-based examples demonstrate the versatility of multimodal platforms, their direct translation to cardiology requires careful adaptation to account for fundamental differences in tissue biology, target accessibility, and therapeutic goals.

The principle of image-guided therapy is powerfully augmented by nanotechnology. Multifunctional nanoparticles can serve as dual-purpose agents: as contrast enhancers for high-sensitivity MRI, PAI, or radionuclide imaging to precisely delineate the target area, and as carriers for therapeutic payloads. Radiolabeled or photosensitizer-loaded nanogels, for example, allow for real-time visualization of drug distribution and treatment efficacy feedback [32]. This closed-loop strategy ensures that therapy is delivered precisely to the intended target and can be adjusted based on immediate imaging feedback.

Applied to ventricular arrhythmias, a multimodal nanotherapeutic strategy could involve: (1) using targeted imaging nanoprobes to map the precise geometry and electrophysiological properties of an arrhythmogenic scar. (2) Employing stimulus-responsive nanocarriers to deliver a cocktail of anti-fibrotic, anti-inflammatory, and gap junction-modulating drugs directly to that scar. (3) Potentially applying nanoparticle-assisted PTT for focal ablation of critical conducting channels within the scar or photothermal neuromodulation of cardiac autonomic nerves, all guided and monitored by the same nanoplatform [33, 34].

Collectively, evidence supports that such multimodal nanotechnology-enabled combination strategies enhance treatment specificity, mitigate systemic toxicity, and can lead to improved therapeutic outcomes by addressing disease pathophysiology through multiple concurrent mechanisms [35]. While challenges in biocompatibility, scalable manufacturing, and regulatory pathways persist [32, 36], the ongoing evolution toward personalized, image-guided, and combinatorial nanomedicine holds significant promise for developing more effective and durable treatments for complex cardiac conditions like ventricular arrhythmias.

The successful clinical implementation of the nanotechnology-based therapies discussed above will require more than technological innovation alone. These sophisticated interventions introduce new complexities into patient care that demand a corresponding evolution in nursing practice and healthcare delivery systems. Nurses will be at the forefront of managing patients receiving nanotherapies, with responsibilities including: (1) pre-administration assessment to identify patient-specific risk factors and contraindications; (2) administration monitoring for infusion-related reactions, acute adverse effects, and device-nanomaterial interactions; (3) post-procedural surveillance for delayed toxicities, including cardiac monitoring for pro-arrhythmic effects; (4) patient education regarding treatment rationale, expected outcomes, and symptom recognition; and (5) coordination with multidisciplinary teams to ensure seamless care transitions. These requirements necessitate the development of specialized nursing competencies, standardized protocols, and integrated care pathways—topics addressed in the following sections.

## Comprehensive nursing strategies for patients with ventricular arrhythmias

### Nursing assessment and individualized care planning

Comprehensive and systematic nursing assessment serves as the cornerstone for developing individualized care plans, particularly for patients with complex conditions such as ventricular arrhythmias who may be candidates for or recipients of nanotechnology-based therapies. This process must extend beyond the evaluation of physical symptoms to integrate a multidimensional examination of the patient’s psychological status, social support system, lifestyle habits, and treatment adherence. For patients receiving nanotherapies, assessment must also include:

- Baseline cardiac function, including detailed electrocardiographic parameters to enable post-treatment comparison.
- Assessment of bleeding risk, hepatic and renal function (critical for nanoparticle clearance).
- Evaluation of potential contraindications (e.g., known hypersensitivity to nanomaterial components).
- Identification of concomitant medications that may interact with nanocarrier systems or payloads.

The use of standardized assessment tools—such as frameworks based on Gordon’s Functional Health Patterns or the interRAI Acute Care assessment system—in conjunction with Electronic Health Record (EHR) systems enables the structured collection and analysis of patient data. This integration provides critical support for the rapid formulation of precise nursing plans. Research indicates that electronic care planning systems incorporating standardized assessments can significantly increase the proportion of patients receiving an individualized plan within 24 h of admission, highlighting the importance of early and systematic evaluation [37].

The core of an individualized care plan lies in the integration of medical, psychological, and social interventions to form a patient-centered care model aligned with the patient’s values and preferences. In the management of ventricular arrhythmias, particularly in the context of novel nanotherapies, this specifically translates into: (1) medication management: nurses must ensure patients understand the purpose, administration, and potential side effects of antiarrhythmic drugs—whether delivered conventionally or via nanocarriers—while monitoring for efficacy and adverse reactions (e.g., QT interval prolongation, arrhythmia exacerbation, infusion reactions). (2) Lifestyle intervention: providing specific guidance on limiting caffeine/alcohol intake, maintaining a balanced diet, engaging in regular aerobic exercise, and managing stress. (3) Psychosocial support: identifying and intervening in anxiety, fear, or depression related to the disease, implanted devices (e.g., ICDs), or participation in novel therapy trials, as these psychological factors can directly affect autonomic balance and trigger or worsen arrhythmias [38–40]. The application of standardized nursing terminologies (e.g., NANDA-I, NIC, NOC) aids in structurally defining nursing problems, interventions, and expected outcomes, thereby enhancing interdisciplinary communication and care continuity [41–43].

**Fig. 2:**
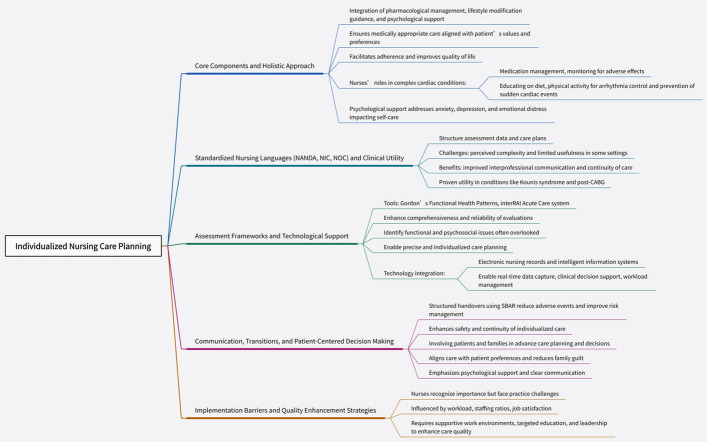
Individualized care planning

The successful implementation of care plans depends on effective communication and a supportive work environment. Utilizing standardized handover frameworks (e.g., SBAR) and involving patients and families in shared decision-making have been shown to significantly improve safety and patient satisfaction [44–47]. Concurrently, nurses’ perception and execution of individualized care are influenced by factors such as workload, staffing levels, and leadership support. Optimizing these factors is key to ensuring high-quality, consistent nursing care [48, 49], illustrated in Fig. 2.

### Electrocardiographic monitoring and complication prevention

Continuous electrocardiographic (ECG) monitoring is a fundamental tool for the early detection, risk stratification, and efficacy evaluation of ventricular arrhythmias. Compared to routine short-duration ECGs, ambulatory Holter monitoring or long-term patch-based ECG monitoring significantly increases the detection rate of paroxysmal and asymptomatic arrhythmias. This is crucial for guiding therapeutic decisions, such as catheter ablation or medication adjustments [50–53]. Advanced monitoring technologies integrated with machine learning algorithms are further enhancing the accuracy and noise resistance of automated analysis for complex arrhythmias [54, 55].

For patients receiving nanotechnology-based therapies, ECG monitoring assumes additional importance as a surveillance tool for potential pro-arrhythmic effects. Nanoparticle–cardiac tissue interactions, while generally intended to be therapeutic, may theoretically alter cardiac electrophysiology through several mechanisms: (1) direct interaction with ion channels; (2) oxidative stress-mediated channel modification; (3) inflammatory modulation of gap junction function, or (4) unintended accumulation in cardiac conduction tissue. Consequently, pre- and post-administration ECG monitoring should be protocolized, with particular attention to:

- Changes in QT interval duration and dispersion.
- New or increased frequency of premature ventricular complexes.
- Development of non-sustained or sustained ventricular tachycardia.
- Alterations in heart rate variability (as a marker of autonomic tone).

In nursing practice, the ultimate goal of monitoring is complication prevention. This requires nurses to possess proficient monitoring skills and risk capabilities: (1) recognizing malignant warning signs: closely observing ECG changes and vigilantly monitoring for precursors of malignant arrhythmias, such as R-on-T premature ventricular complexes, short runs of ventricular tachycardia, and significant QTc interval prolongation. (2) Preventing thromboembolism: for patients with concurrent atrial fibrillation or cardiac dysfunction, strict management of anticoagulation therapy and monitoring for signs of bleeding or thrombosis are essential. (3) Managing hemodynamics: closely observing the patient’s consciousness, blood pressure, urine output, and signs of heart failure (e.g., dyspnea, edema) to promptly identify and manage hemodynamic compromise caused by arrhythmias [56, 57]. Standardized monitoring protocols and regular training form the basis for ensuring monitoring quality and reducing human error [55, 58, 59].

### Health education and patient self-management

Empowering patients and their families is pivotal for effective long-term disease management [60]. Health education for ventricular arrhythmias should be a structured, individualized, and continuous process encompassing [61]: (1) disease knowledge: explaining the mechanism of arrhythmias, common triggers, and symptoms in layperson’s terms. (2) Treatment adherence: emphasizing the importance of medication compliance, regular follow-ups, and the maintenance of implanted devices (e.g., ICDs, pacemakers). (3) Lifestyle management: providing specific guidance on diet (e.g., sodium restriction, maintaining electrolyte balance), exercise (avoiding excessive intensity), smoking cessation, alcohol limitation, and stress management. (4) Symptom recognition and emergency response: educating patients to recognize warning symptoms such as palpitations, dizziness, and presyncope, and to know when and how to seek urgent medical help.

For patients receiving or considering nanotechnology-based therapies, education must be expanded to include: (5) nanotherapy-specific information: explanation of the rationale for nanocarrier delivery, expected benefits (e.g., reduced side effects, targeted action), and potential short- and long-term risks in age-appropriate, understandable language. (6) Post-treatment expectations: description of monitoring requirements, potential immediate sensations (e.g., warmth during photothermal therapy), and symptoms that should prompt medical contact. (7) Clinical trial considerations: for patients enrolled in investigational protocols, detailed explanation of the research nature of the treatment, unknown risks, and the importance of adherence to follow-up schedules.

Educational methods should be flexible and diverse, combining face-to-face instruction, written materials, and digital platforms (e.g., apps, social media groups) to meet the needs and preferences of different patients [62–65]. As the primary providers of health education, nurses must continuously strengthen their own professional knowledge and communication skills through ongoing professional development, including training in emerging nanotechnologies. Effective education significantly improves patients’ health literacy, self-management capabilities, and quality of life, ultimately reducing readmission rates [60, 61, 66–69].

### Psychological nursing and social support

A well-established vicious cycle exists between psychological stress and ventricular arrhythmias. Emotions such as anxiety and depression can increase the risk of arrhythmic events by activating the sympathetic nervous system and lowering the ventricular fibrillation threshold. Therefore, integrating psychosocial assessment and support into routine care is indispensable. This is particularly important for patients receiving novel nanotherapies, who may experience additional anxiety related to the experimental nature of treatment, uncertainty about long-term outcomes, and the burden of intensive monitoring protocols. Nursing interventions include: (1) systematic psychological assessment: regularly screening patients for anxiety, depression, and disease-related psychological distress using standardized scales. (2) Providing psychological interventions: implementing individual or group psychological counseling, cognitive-behavioral therapy, and relaxation training (e.g., mindfulness, diaphragmatic breathing) to help patients develop coping strategies and alleviate psychological burden. Research shows that comprehensive interventions integrating psychological care can effectively reduce arrhythmia recurrence and improve patients’ quality of life [70, 71]. (3) Building social support networks: encouraging family involvement, connecting patients with peer support groups, and utilizing community resources to reduce feelings of isolation and helplessness. Strong social support has been identified as a protective factor for improving psychological prognosis and treatment adherence [72–76].

In summary, the nursing care for patients with ventricular arrhythmias must adopt a comprehensive strategy integrating the biopsychosocial model. This involves developing individualized plans based on systematic assessment, preventing complications through precise monitoring, empowering patients via effective education—including nanotechnology-specific content—and providing holistic psychosocial support. Only through this integrated approach can patient outcomes be optimized in all dimensions, quality of life enhanced, and the perfect synergy between advanced technology and humanistic care in high-level medical practice be ultimately achieved [77–79].

## Application of standardized management in the care of resistant cardiac arrhythmias

### Standardization of nursing process construction

The standardization of nursing processes is central to ensuring the quality and safety of care for patients with ventricular arrhythmias, particularly when integrating advanced therapies such as multimodal nanotechnology. Its core involves the development of evidence-based, scientific nursing operating procedures that encompass all stages from initial assessment and intervention to follow-up. These protocols must be meticulously designed to address the unique complexities of arrhythmia management, including the monitoring of adverse events, medication administration, and patient education. For nanotechnology-based therapies, standardized protocols must also specify:

- Pre-administration assessment parameters (e.g., cardiac function, renal/hepatic clearance capacity, pregnancy status).
- Administration procedures (e.g., infusion rates, pre-medication requirements, monitoring during infusion).
- Post-administration surveillance protocols (e.g., ECG monitoring frequency, laboratory testing intervals, duration of observation).
- Adverse event identification and management algorithms specific to nanomaterial-related reactions (e.g., infusion reactions, complement activation-related pseudoallergy, delayed hypersensitivity).
- Documentation requirements for nanotherapy administration and outcomes.

For instance, comprehensive nursing interventions have been shown to significantly improve cardiac function indices, reduce arrhythmia episodes, and enhance sleep quality and psychological well-being in patients with post-acute myocardial infarction arrhythmias [80]. This underscores the importance of standardizing protocols that incorporate holistic care elements, thereby ensuring consistency and safety in nursing delivery.

In modern nursing practice, the integration of information management systems is paramount for enabling dynamic monitoring and continuous optimization of workflows. Electronic health records and telemetry data systems allow for real-time tracking of patient status, facilitating the early detection of arrhythmias and timely intervention. For example, automated algorithms developed for detecting atrial fibrillation from continuous ECG data demonstrate how informatics can enhance accuracy and efficiency beyond traditional nursing documentation and diagnostic coding methods [81]. These systems minimize human error and provide objective data to support clinical decision-making. Furthermore, novel patch-based continuous cardiac rhythm monitoring devices have proven the feasibility of improving monitoring fidelity in non-ICU settings, although challenges such as noise interference remain to be addressed [82]. Integrating such technologies into standardized nursing protocols requires staff training and process adaptation but promises significant improvements in surveillance capability and patient safety.

The combination of standardized nursing procedures and advanced information systems also supports quality improvement initiatives. For example, evidence-based practice methodologies have successfully reduced the incidence of complications such as amiodarone-related phlebitis by standardizing intravenous (IV) site assessment frequency, vein selection criteria, and the use of grading tools for early identification [83]. This practice exemplifies how structured protocols and data-driven monitoring can mitigate risks associated with pharmacological therapies commonly used in arrhythmia management. Concurrently, historical and contemporary nursing practices in the management of devices such as pacemakers and implantable cardioverter-defibrillators indicate that protocol-driven care is necessary for the safe and effective handling of complex devices [84]. The evolution of nursing standards alongside technological advancements continually reinforces the necessity of process standardization for maintaining high-quality care.

### Multidisciplinary collaboration model

A multidisciplinary collaboration model is essential for managing ventricular arrhythmias, particularly as therapeutic options expand to include nanotechnology-based interventions. This model integrates expertise from cardiology (including electrophysiology), nursing, pharmacy, biomedical engineering, and rehabilitation medicine to optimize patient outcomes. Cardiologists and electrophysiologists provide precise diagnoses and interventional strategies, including appropriate selection of patients for nanotherapy trials or established treatments. Nursing professionals ensure continuous monitoring, patient-centered care, and serve as the primary coordinators of the multidisciplinary team. Clinical pharmacists contribute by tailoring antiarrhythmic drug regimens, managing potential drug interactions, and providing expertise on nanocarrier–pharmacology interactions. Biomedical engineers offer critical insights into nanomaterial design, device integration, and troubleshooting. Rehabilitation specialists facilitate functional recovery and secondary prevention.

Establishing structured communication channels across these disciplines enhances information exchange, aligns treatment goals, and mitigates risks associated with fragmented care. In the context of applying multimodal nanotechnology for ventricular arrhythmia treatment, this collaborative approach is essential for:

- Developing and approving standardized protocols for nanotherapy administration.
- Establishing competency requirements and training programs for nursing staff.
- Creating patient education materials that accurately reflect both technological and clinical aspects of care.
- Coordinating follow-up monitoring and data collection for outcomes assessment.
- Facilitating rapid response to unexpected adverse events through pooled expertise.

This collaborative approach fosters comprehensive management, thereby improving both clinical efficacy and patient quality of life.

### Quality control and continuous improvement

Establishing robust quality control measures and fostering a culture of continuous improvement are critical components of nursing management, especially in complex clinical scenarios such as ventricular arrhythmia management and the application of multimodal nanotechnology. The development of a nursing quality evaluation indicator system provides a structured framework for systematically assessing care delivery, identifying gaps, and implementing targeted interventions. For nanotechnology-based therapies, quality indicators should extend beyond conventional metrics to include:

- Adherence to nanotherapy administration protocols.
- Completeness of pre-treatment assessment documentation.
- Timeliness and appropriateness of post-treatment monitoring.
- Incidence and management of nanotherapy-related adverse events.
- Patient comprehension of nanotherapy-specific education.
- Staff competency in nanotherapy-related skills and knowledge.

For instance, the European Society of Cardiology has developed comprehensive quality indicators encompassing multiple domains of care for patients with ventricular arrhythmias, including structural framework, screening, risk stratification, patient education, pharmacological treatment, device therapy, catheter ablation, and clinical outcomes [85]. These indicators serve as benchmarks for evaluating nursing performance and patient outcomes, enabling regular quality audits and feedback loops that promote accountability and transparency in care delivery. Periodic audits based on these indicators help identify deviations from established standards and prompt corrective actions, thereby enhancing patient safety and care effectiveness.

Integrating evidence-based medicine into nursing practice is vital for continuous quality improvement. Nursing protocols and care pathways must be dynamically updated to reflect the latest clinical guidelines and research findings, ensuring patients receive care grounded in the best available evidence. For example, updates in pharmacological regimens, device implantation criteria, and ablation techniques for managing ventricular arrhythmias necessitate corresponding revisions in nursing care plans to optimize patient monitoring, education, and complication prevention [86]. As evidence emerges regarding the safety and efficacy of nanotherapies, protocols must be promptly updated to incorporate new knowledge. Adopting a cyclical quality improvement model—comprising planning, implementation, evaluation, and refinement—supports the sustained advancement of nursing practices. This model encourages multidisciplinary collaboration, leveraging insights from cardiologists, electrophysiologists, biomedical engineers, and nursing specialists to develop interventions that address evolving clinical challenges.

Beyond formal quality indicators and evidence-based updates, the use of advanced technologies such as implantable loop recorders and nanotechnology-based therapeutics demands specialized nursing competencies and vigilance. Nurses play a pivotal role in device management, patient education regarding device function and symptom reporting, and the early detection of adverse events [87, 88]. Continuous training and competency assessments ensure nursing staff remain proficient in handling these sophisticated modalities, contributing to improved patient outcomes and satisfaction.

Furthermore, quality control should extend beyond clinical procedures to encompass holistic aspects of care, including psychosocial support, lifestyle modification counseling, and adherence promotion. For patients with arrhythmias, particularly those with implanted devices or undergoing novel therapies like stereotactic arrhythmia radioablation or nanotherapy, comprehensive nursing care addressing emotional well-being and quality of life is indispensable [89, 90]. Continuous improvement efforts should incorporate strategies to optimize these dimensions, guided by patient-centered outcomes and emerging evidence.

### Patient safety management

Effective patient safety management is a critical component in the clinical application of multimodal nanotechnology for treating ventricular arrhythmias. Implementing comprehensive risk assessment protocols and preventive measures is essential to reduce the incidence of medical errors and adverse events. Given that nanotechnology-based therapies often involve novel drug delivery systems (e.g., liposomes, dendrimers, polymeric nanoparticles) designed to minimize cardiotoxicity, a thorough evaluation of patient-specific risk factors must precede treatment initiation. This approach not only anticipates potential complications but also facilitates tailored interventions to enhance therapeutic safety. For instance, nanocarriers have been shown to alter the pharmacokinetics and pharmacodynamics of cardiotoxic drugs, thereby mitigating off-target effects and preserving myocardial tissue integrity [27]. Nevertheless, vigilance is required to monitor for unexpected adverse reactions or interactions that may arise from these innovative modalities, including:

- Infusion-related reactions: nanoparticles may activate the complement system, leading to complement activation-related pseudoallergy (CARPA), characterized by flushing, dyspnea, hypotension, and chest pain. Protocols must include pre-medication strategies (e.g., antihistamines, corticosteroids) and graded infusion rates for naïve patients.
- Pro-arrhythmic effects: as discussed in "Electrocardiographic monitoring and complication prevention" section, nanoparticle–cardiac tissue interactions may theoretically alter cardiac electrophysiology. Continuous ECG monitoring during and after initial administrations is essential, with predefined criteria for treatment interruption.
- RES accumulation and delayed toxicity: nanoparticles accumulate in the liver, spleen, and bone marrow, with potential for delayed organ toxicity. Long-term follow-up protocols should include periodic assessment of hepatic and renal function, complete blood counts, and cardiac imaging.
- Drug–nanoparticle interactions: nanocarriers may alter the disposition of concomitant medications, necessitating therapeutic drug monitoring for narrow therapeutic index drugs.

In addition to risk assessment, strengthening key safety procedures such as patient identification, medication management, and infection control is paramount. Accurate patient identification prevents administration errors, particularly when complex nanomedicine regimens are involved. Medication safety is especially critical, as the integration of nanotechnology may alter drug formulations and dosing schedules, necessitating meticulous oversight to avoid dosing errors or drug incompatibilities. Moreover, infection control protocols must be rigorously enforced, particularly for intravenous therapies. For example, intravenous administration of amiodarone has been associated with phlebitis. An evidence-based quality improvement project demonstrated that increasing the frequency of IV site assessments, selecting appropriate veins, and employing standardized grading tools could significantly reduce the incidence and severity of amiodarone-related phlebitis by up to 48% within 6 months post-intervention [83]. This example underscores the importance of continuous monitoring and adherence to safety protocols to minimize complications associated with intravenous drug delivery—a route frequently used in nanomedicine applications.

**Fig. 3:**
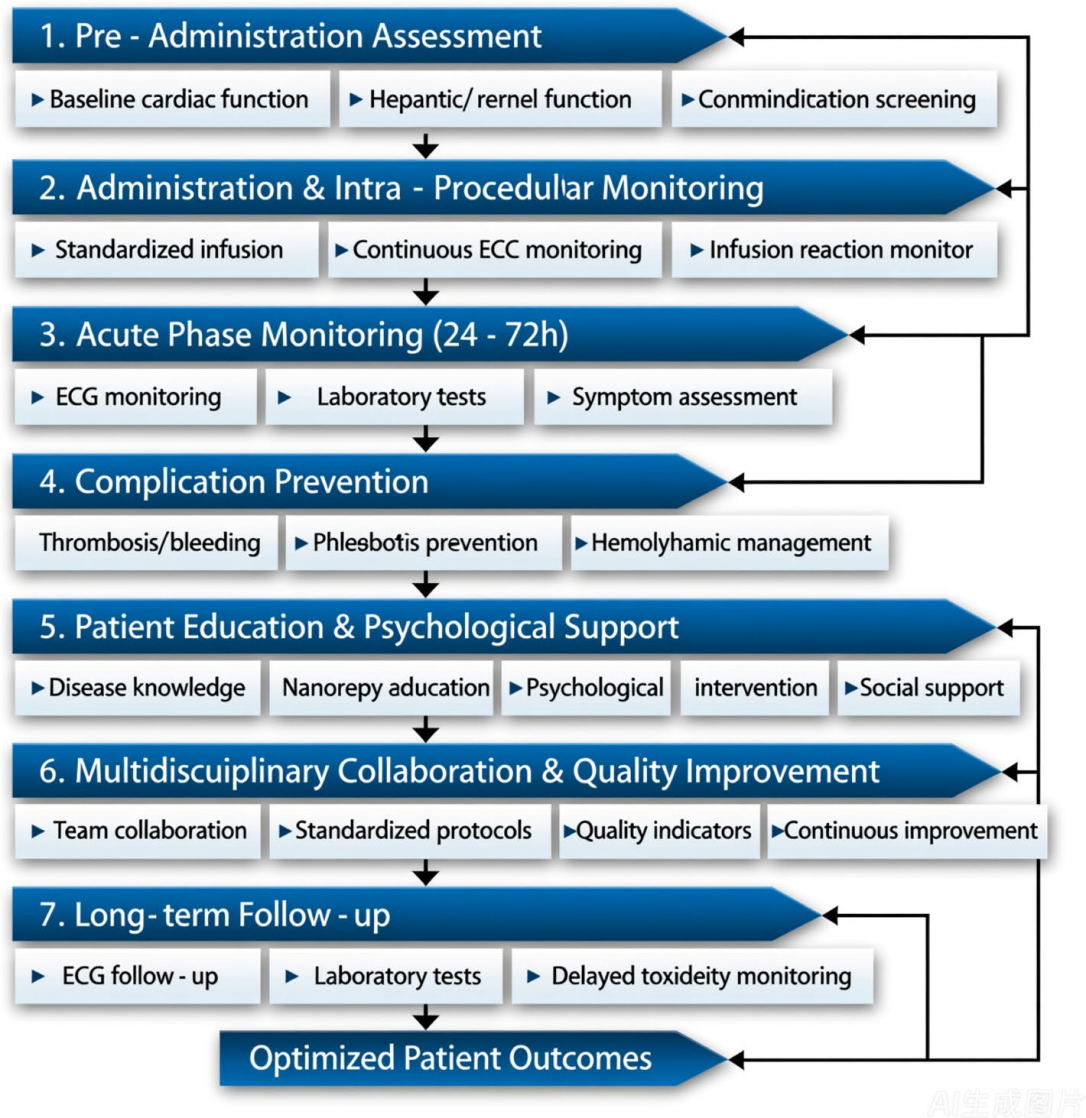
Integrated care pathway from nanotherapy administration to nursing follow-up

Finally, integrating multidisciplinary guidelines based on current evidence is vital for standardizing safety practices. These guidelines should encompass detailed procedures for risk stratification, patient monitoring, and adverse event reporting, fostering a culture of safety and accountability. The rapid evolution of nanotechnologies necessitates ongoing education and training for healthcare professionals to stay abreast of emerging risks and best practices. By embedding evidence-based safety measures into routine clinical workflows, healthcare providers can optimize patient outcomes and reduce harm, as illustrated in Fig. 3.

## Conclusion

In summary, the application of multimodal nanotechnology in the diagnosis and treatment of ventricular arrhythmias signifies a paradigm shift in cardiovascular medicine. This theranostic platform offers substantial enhancements in intervention precision and efficacy through targeted delivery mechanisms and a marked reduction in systemic adverse effects. Its capacity to integrate real-time, high-resolution diagnostic imaging with therapeutic functionality presents a compelling strategy to address the limitations of conventional approaches, which are often constrained by suboptimal specificity and significant procedural risks. However, it is imperative to recognize that the field is at an early stage, with much of the foundational evidence derived from oncology and requiring rigorous validation in cardiovascular models. Critical cardiac-specific challenges—including the absence of EPR effects, the anatomical barriers posed by continuous myocardial capillaries, the need for active transcytosis-based delivery, and the potential for pro-arrhythmic effects—must be systematically addressed through preclinical and clinical investigation. The transition of these sophisticated technologies from bench to bedside necessitates rigorous validation through controlled clinical trials to definitively establish their safety, reproducibility, and long-term cost-effectiveness.

Concurrently, comprehensive and individualized nursing care remains an indispensable pillar in the holistic management of patients with ventricular arrhythmias. The introduction of nanotechnology-based therapies will fundamentally impact nursing practice, requiring new competencies in nanotherapy administration, monitoring for novel adverse effect profiles, patient education regarding these innovative treatments, and coordination within expanded multidisciplinary teams. Care plans that proactively integrate psychological support, structured patient education (including nanotechnology-specific content), and meticulous symptom monitoring are critical not only for facilitating physiological recovery but also for mitigating the emotional and psychosocial burdens inherent to chronic cardiac conditions and participation in novel therapy trials. This integrated, patient-centered approach is fundamental to improving treatment adherence, minimizing complications, and enhancing overall quality of life.

Furthermore, the implementation of standardized management protocols and the cultivation of robust multidisciplinary collaboration are foundational to ensuring consistent care quality and maximizing therapeutic synergy. Effective convergence of expertise from cardiology, biomedical engineering, nursing, and pharmacy fosters an environment conducive to seamless knowledge exchange, protocol refinement, and continuous quality improvement. This collaborative model not only optimizes clinical workflows but also accelerates the translational pipeline, promoting more scientific and standardized management of ventricular arrhythmias. Challenges persist in aligning diverse professional perspectives and institutional resources, underscoring the need for sustained efforts in refining coordination frameworks and interdisciplinary training.

Looking forward, future endeavors should prioritize the translational pathway of nanotechnology, with focused research on biocompatibility, scalable manufacturing, regulatory navigation, and comprehensive long-term safety evaluation—including rigorous assessment of pro-arrhythmic potential. Parallel innovation in nursing science is equally critical, emphasizing the adoption of digital health tools, personalized intervention models, and outcome-driven care frameworks to further improve patient prognosis. By strategically converging technological progress with compassionate, evidence-based nursing and robust systemic support, the field can advance toward a more integrated, effective, and sustainable paradigm for ventricular arrhythmia care. This comprehensive strategy holds the promise of not only deepening pathophysiological understanding but also delivering meaningful clinical impact, ultimately improving survival and quality of life for this high-risk patient population.

## Future research directions and development trends

The future integration of nanotechnology into precision medicine for ventricular arrhythmia management is poised to evolve along several pivotal and interconnected avenues. A primary focus will be the advancement of multi-targeted therapeutic strategies and integrated theranostic platforms. Leveraging the unique modularity of nanomaterials, future systems will be engineered to co-deliver diverse therapeutic payloads—such as specific ion channel blockers, anti-fibrotic agents, gene-editing tools, and immunomodulators—in a spatiotemporally controlled manner. This approach aims to concurrently modulate the electrophysiological, structural, and inflammatory substrates of arrhythmogenesis, thereby overcoming the limitations of single-mechanism drugs. Furthermore, the convergence of nanotechnology with advanced bioelectronics, particularly implantable and bioresorbable bioelectrodes, will facilitate the development of closed-loop systems. These “smart” implants could provide real-time electrophysiological mapping, detect arrhythmic triggers with high sensitivity, and respond with precisely calibrated electrical neuromodulation or localized drug release, creating a dynamic and adaptive treatment ecosystem [27, 91].

Progress in this field will be fundamentally driven by interdisciplinary convergence and data-driven design. The complexity of arrhythmia pathophysiology necessitates deep collaboration across nanotechnology, systems biology, computational modeling, and clinical electrophysiology. The aggregation and sophisticated analysis of multi-omics data (genomic, proteomic, metabolomic) alongside detailed clinical phenotyping will enable the creation of predictive digital twins and patient-specific treatment algorithms. This big-data paradigm will not only guide the rational design of next-generation nanotherapeutics with optimal targeting and release kinetics but also refine patient stratification for tailored interventions. This data-centric approach extends to optimizing comprehensive care protocols, ensuring that technological innovations are effectively translated into standardized, high-quality nursing and management practices. The exploration of nanotechnology to enhance the delivery and efficacy of bioactive natural compounds represents a promising synergy between traditional pharmacological insights and modern nano-engineering [92].

Cardiac-specific translational research priorities must include:

- Development of active targeting strategies optimized for myocardial delivery: given the absence of EPR effects in cardiac tissue, research must focus on receptor-mediated transcytosis mechanisms, with identification of cardiac-specific or pathology-specific targets (e.g., AT1R in scars, VCAM-1 in inflammation, tenascin-C in fibrosis) and optimization of ligand density, affinity, and multivalency.
- Investigation of nanoparticle–cardiac electrophysiology interactions: systematic evaluation of nanomaterial effects on ion channel function, action potential duration, conduction velocity, and arrhythmia susceptibility in relevant preclinical models (e.g., human induced pluripotent stem cell-derived cardiomyocytes, ex vivo perfused hearts, large animal models).
- Optimization of light delivery systems for deep myocardial targets: engineering of catheter-based intracardiac light delivery systems, development of implantable miniaturized light sources, and investigation of ultrasound-mediated or photoacoustic-guided targeting to overcome penetration and motion challenges.
- Long-term safety assessment in chronic models: evaluation of nanoparticle biodistribution, retention, degradation, and chronic toxicity in large animal models with extended follow-up (≥1 year), with particular attention to cardiac function, coronary artery integrity, and pro-arrhythmic potential.

Ultimately, translating these innovations into routine clinical practice hinges on overcoming persistent translational challenges. Key among these is enhancing the long-term biocompatibility and functional integration of implantable nanodevices. Research must prioritize novel surface engineering, biomimetic coatings, and the use of biodegradable materials to minimize foreign body responses and ensure device longevity. Concurrently, establishing scalable and reproducible manufacturing processes, navigating rigorous regulatory pathways for combination products (device + drug), and conducting robust long-term safety and outcome studies are imperative. By systematically addressing these barriers through coordinated multidisciplinary efforts, the field can move toward realizing the full potential of nanotechnology to enable a new era of precise, effective, and personalized management for ventricular arrhythmias.

## Data Availability

No datasets were generated or analyzed during the current study.
